# Nationwide patterns of hydroxychloroquine dosing and monitoring of retinal toxicity in patients with systemic lupus erythematosus

**DOI:** 10.1038/s41598-023-34022-0

**Published:** 2023-05-04

**Authors:** Jae-Eun Lee, Dal Ri Nam, Yoon-Kyoung Sung, Yu Jeong Kim, Sun-Young Jung

**Affiliations:** 1 Department of Global Innovative Drugs and College of Pharmacy, Chung-Ang University, Seoul, 06974 Republic of Korea; 2 Department of Rheumatology, Hanyang University Hospital for Rheumatic Diseases, Seoul, 04763 Republic of Korea; 3 Department of Ophthalmology, Hanyang University Hospital, Seoul, 04763 Republic of Korea

**Keywords:** Epidemiology, Rheumatic diseases, Epidemiology

## Abstract

This study identified trends in hydroxychloroquine (HCQ) prescription and retinopathy screening in patients with systemic lupus erythematosus (SLE) according to clinical practice guidelines to minimise the risk of HCQ retinopathy. We used data from patients diagnosed with SLE between 2004 and 2019 from the National Health Insurance Service in Korea. To assess trends of daily dose per actual body weight (ABW), we performed an interrupted time-series analysis and identified effects after revision of guidelines. Among 38,973 patients with SLE, 28,415 (72.9%) were prescribed HCQ from 2004 to 2019. The proportion of patients using HCQ among SLE patients was 63% in 2004 and increased to 76% in 2019. The median daily dose per ABW for HCQ users decreased from 5.88 mg/kg in 2004 to 3.98 mg/kg in 2019, and from 5.45 mg/kg in 2005 to 4.17 mg/kg in 2019 for HCQ new users. The annual implementation rate of screening tests among HCQ new users increased from 3.5% in 2006 to 22.5% in 2019. Study results indicated that HCQ dosing management was adequate based on the revised guidelines. Although the implementation rate of retinal screening has increased, it is necessary to enhance awareness of retinal screening in clinical settings.

## Introduction

Hydroxychloroquine (HCQ) is recommended for all patients with systemic lupus erythematosus (SLE) and has wide-ranging benefits^1^. Although HCQ has remained the treatment option for SLE for over half a century, the prevalence of HCQ retinopathy, a rare but serious adverse event, has been reported. A recent study on 310 patients in South Korea revealed that the prevalence of HCQ retinopathy was 2.9% in overall HCQ users, and 5.2% with five years or longer HCQ use, when using spectral-domain optical coherence tomography (SD-OCT), automated visual field test or fundus autofluorescence^2^. A previous study in the US reported a 7.5% prevalence when using central visual field testing or SD-OCT among 2,361 patients who received HCQ for at least 5 years^3^. While the mechanism of HCQ-induced retinal toxicity is not fully understood, a possibility that HCQ binds to melanin in the retinal pigment epithelium (RPE) and induces RPE degeneration, has been suggested^4^. The most severe aspect of retinal toxicity is irreversible vision-threatening toxicity^5^.

To reduce the retinal toxicity of HCQ, the American Academy of Ophthalmology (AAO) revised its guidelines twice. The 2011 AAO guidelines were revised to recommend daily HCQ doses < 6.5 mg/kg of ideal body weight (IBW)^6^. In 2014, a US population-based study established that actual body weight (ABW)-based dosing predicted rates of toxic retinopathy more accurately than IBW-based dosing, and receiving daily doses of > 5.0 mg/kg of ABW increased the risk of retinal toxicity^3^. Following these findings, the AAO revised its guidelines to recommend daily doses < 5.0 mg/kg of ABW for retinal safety in 2016^7^.

The AAO guidelines also recommended a baseline examination within the first year of starting HCQ and an annual screening after five years of use. Automated visual fields and SD-OCT are used for routine primary screening, and multifocal electroretinogram and fundus autofluorescence can be additional useful screening tests^7^. Regular screening can help prevent serious retinal damage by early detection of retinal changes and stopping HCQ before significant vision loss^8^.

Several studies discovered a sharp decline in HCQ dosing from 2007 to 2016 and suggested the current screening practices for long-term users^5,9^. Despite these results, literature on the annual trends of HCQ prescription dosage and screening rates before and after the 2016 guidance revision is lacking.

To identify the use and management of HCQ in patients with SLE, we evaluated the proportion of patients using HCQ among SLE patients. We also assessed the daily prescription dose of HCQ and implementation rate of retinal screening from 2004 to 2019 to identify the annual trends in HCQ prescriptions and retinopathy screening according to updates of clinical guidelines, including 2011 and 2016.

## Methods

### Data source

This study used customised health information data (CHID, NHIS-2020–1-453) from 2004 to 2019 provided by the National Health Insurance Service in Korea (NHIS). Korea has a single medical insurance claim system, with more than 97% of the population registered with NHIS^10^. The dataset was extracted by restricting them to a specific study population from the original claim data. This data contains patient demographic information, diagnostic codes with the International Classification of Diseases, tenth revision (ICD-10), prescription information and health examination information.

### Study population

The study population was defined as patients diagnosed with SLE at least once in the data between 2004 and 2019. SLE was defined by the ICD-10 code M32 and Individual Copayment Beneficiaries Programme (ICBP) code V136. The ICBP is for patients with rare and intractable diseases to reduce the burden of medical expenses^11^. Among them, HCQ users were defined as patients prescribed HCQ for SLE between 2004 and 2019. We also defined HCQ new users to assess the implementation rate of baseline retinal screening examinations for patients who initiated HCQ as suggested in the AAO guidelines^7^. HCQ new users were defined as patients first prescribed HCQ for SLE between 2005 and 2019, except in the first year.

For analysing of the trends of daily prescription doses per ABW, we restricted the study to patients who underwent a national health screening with available information on weight within one year before or after the prescription of HCQ.

### Variables

The present study defined the prescription of HCQ using the Anatomical Therapeutic Chemical (ATC) classification system, which recorded the drug code in the claim database. Sex, age and comorbidities were used to identify drug utilisation patterns by patient characteristics. As comorbidities, we included renal and retinal/macular diseases, suggested as risk factors for retinal toxicity in the guidelines^7^. Renal disease was defined as chronic kidney disease (CKD; stage ≥ 3) (2011 ~ 2019, N18.3, N18.4, N18.5; 2008 ~ 2010, N18.0, N18.8; 2004 ~ 2007, N18.0) by ICD-10 code, and retinal/macular disease was defined as H3 by ICD-10 code (Supplementary Table 1)^2,12^. Comorbidity information was obtained within one year from the first prescription date for each year^9^.

To assess the change in daily prescription dose, the dose was presented as a weight-based dose according to the guidelines recommended for the maximum dose per ABW. We used all available prescriptions of HCQ for patients with ABW information. The daily prescription dose per ABW was calculated using the weight information (unit, kg) of national health screening data on the date closest to the prescription date as ABW. Individual national health screening is conducted every two years in Korea; therefore, if each patient’s information on weight was unavailable in the year of the prescription date, we identified the weight information in the national health screening within one year before and after the prescription date.

As recommended retinal screening tests, we included both primary screening tests and other recommended tests suggested in the AAO guidelines. We defined automated visual field and SD-OCT as 'primary screening tests' and electroretinography and fundus autofluorescence as 'other recommended tests'^7^. Retinal screening tests were defined using procedure codes of the Health Insurance Review and Assessment Service (Supplementary Table 2).

### Statistical analysis

We performed an interrupted time-series (ITS) analysis to determine the effectiveness of population-level intervention implemented at clear time points^13^. We assessed the immediate effects after the revision of the guidelines and half-yearly trends of daily prescription doses for patients with SLE from 2004 to 2019. The equation used in the ITS model is as follows:

$${Y}_{t}={\beta }_{0}+{\beta }_{1}\times {TIME1}_{t}+{\beta }_{2}\times {GUIDELINE1}_{t}+{\beta }_{3}\times {TIME2}_{t}+{\beta }_{4}\times {GUIDELINE2}_{t}+{\beta }_{5}\times {TIME3}_{t}+{e}_{t}$$

In this model, Y_t_ is the median daily prescription dose; *t* ranges from 2004 to 2019 on a semi-annual basis; β_0_ is the baseline intercept; β_1_ is the slope of the daily prescription dose until the revision of the guidelines in 2011; β_2_ is the change in the level of the outcome, which is the dose after the 2011 revision of guidelines; β_3_ is the difference between pre- and post-2011 guideline slopes until the revision of the guideline in 2016; β_4_ is the change in the level of the outcome, which is the dose after the 2016 revision of guidelines; β_5_ is the difference between pre- and post-2016 guidelines slopes; e_t_ is the error estimation. The time unit in this model was half-years, and regression coefficients were provided with 95% CI and p-values.

We also analysed annual trends in the implementation rate of retinal screening among HCQ new users. The rate of patients who underwent at least one of the ‘recommended screening tests’ was obtained, assessing 1) within a year from the date of first HCQ prescription and 2) within a year following five-year HCQ use. Annual trends of visiting rates to the ophthalmology department within a year from HCQ new user, both including and excluding patients with retinal/macular disease in the previous year from the prescription date, were also analysed.

The Kruskal–Wallis test was performed to test significant difference among the median daily dose per ABW for each period of 2005–2010, 2011–2015 and 2016–2019. The Cochran-Armitage trend test was used to evaluate the statistical significance of trends of implementation rate of retinal screening tests and visiting rates to the ophthalmology department from 2006 to 2019. A p-value of < 0.05 was considered statistically significant for all results. All analyses were performed using SAS 9.4 (SAS Institute, Cary, NC, USA).

### Ethics statement

The study protocol was approved by the Institutional Review Board of Chung-Ang University (IRB number:1041078–202,003-HR-064–01). Informed consent was waived by the Institutional Review Board of Chung-Ang University, because this study analysed anonymous claims data provided for research by the Korean National Health Insurance Service. All methods were performed in accordance with the relevant guidelines and regulations.

## Results

### Characteristics of the study population

**Figure 1:**
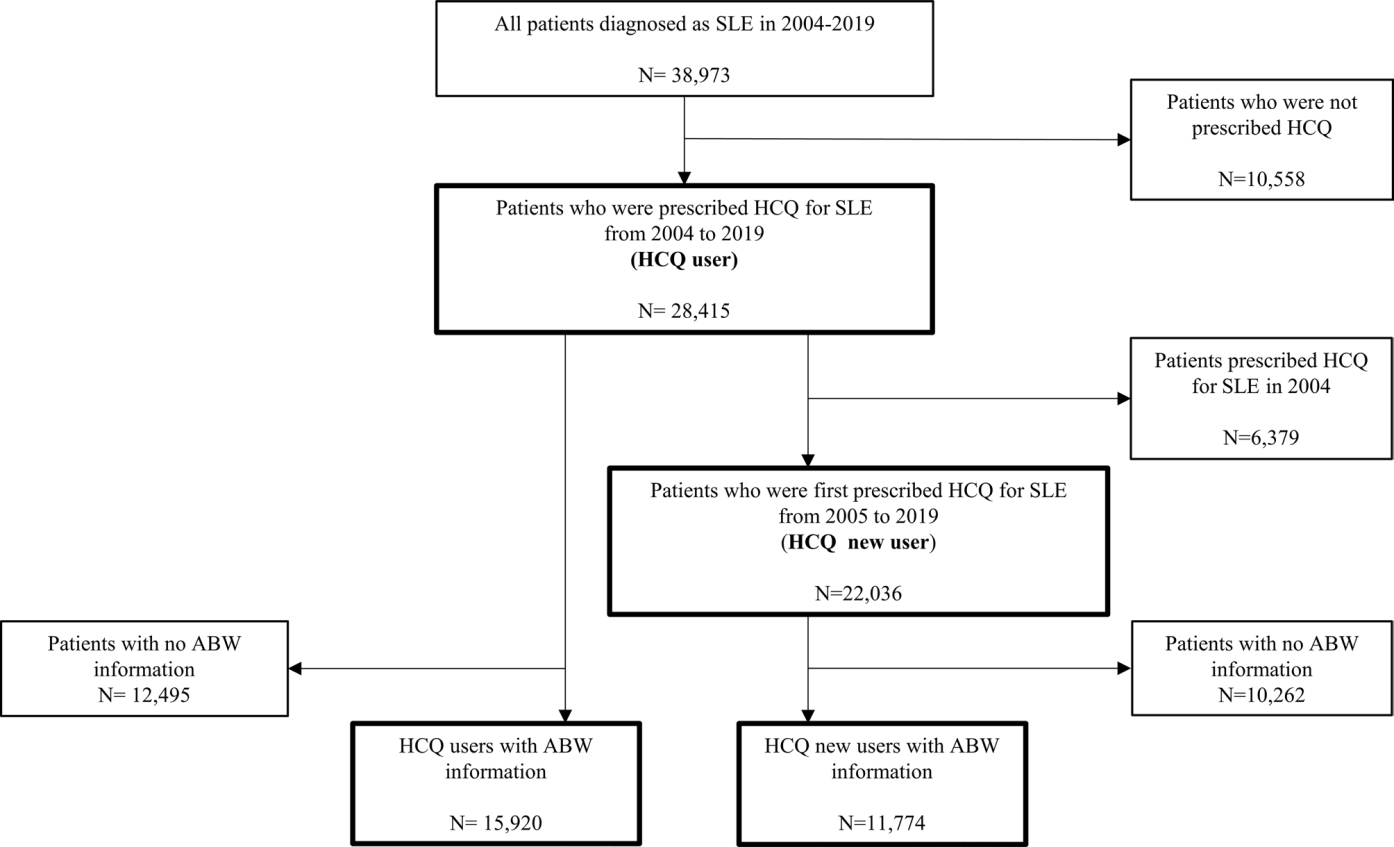
Flowchart of study population. HCQ, hydroxychloroquine; SLE, systemic lupus erythematosus.

Among 38,973 patients with SLE from 2004 to 2019, we defined 28,415 HCQ users from 2004 to 2019 (2004, N = 6,379; 2019, N = 16,578) and 22,036 HCQ new users from 2005 to 2019 (2005, N = 1,817; 2019, N = 1,455). After excluding patients with no ABW information, 15,920 HCQ users with ABW information and 11,774 HCQ new users with ABW information were identified (Fig. 1).

Characteristics of patients prescribed HCQ for SLE and ABW information is available are listed in Table 1. Females comprised 91% of HCQ users and 90% of HCQ new users. The median age was 44 years and 43 years in overall HCQ users and HCQ new users, respectively. About 1% of HCQ users had CKD (≥ stage 3), and more than 12% of HCQ users had retinal/macular disease. The median ABW was about 55 kg in both HCQ users and new users with ABW information. Characteristics of HCQ users and new users by year are identified (Supplementary Table 3).

**Table 1:** Characteristics of patients prescribed HCQ for SLE and with ABW information (HCQ users, 2004 to 2019; HCQ new users, 2005 to 2019).

	HCQ users with ABW information (n = 15,920)	HCQ new users with ABW information (n = 11,774)
Age, years		
Median (min, max)	44 (18, 95)	43 (10, 95)
Mean (SD)	44.53 (12.46)	43.70 (13.51)
≥ 40 years, n (%)	10,962 (68.86)	7,112 (60.40)
Sex		
Women, n (%)	14,450 (90.77)	10,600 (90.03)
Risk factors		
CKD (≥ stage 3), n (%)	130 (0.82)	105 (0.89)
Retinal/macular disease, n (%)	2,153 (13.52)	1,603 (13.61)
ABW, kg*		
Median (min, max)	55 (28, 126)	55.3 (28, 126)
Mean (SD)	56.86 (9.78)	57.16 (9.97)
0 < ≤ 45, n (%)	1,251 (7.86)	885 (7.52)
45 < ≤ 55, n (%)	6,856 (43.07)	4,969 (42.20)
55 < ≤ 65, n (%)	5,233 (32.87)	3,898 (33.11)
> 65, n (%)	2,580 (16.21)	2,022 (17.17)

CKD, chronic kidney disease; ABW, actual body weight.

*ABW was calculated based on the nearest measurement from the first prescription of HCQ during the study period.

### Trend in the prevalence of HCQ use in patients with SLE

**Figure 2:**
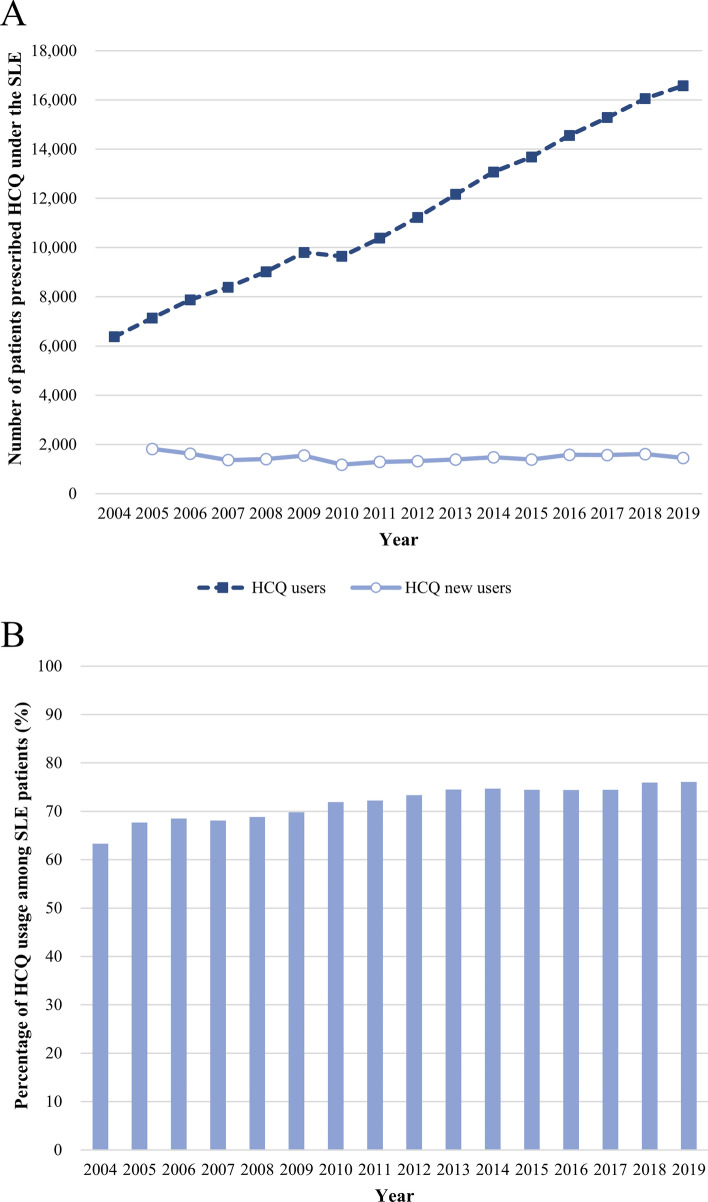
The number of patients prescribed HCQ per year under the diagnosis of SLE (**A**) and the proportion of patients prescribed HCQ among patients with SLE (**B**). HCQ, hydroxychloroquine; SLE, systemic lupus erythematosus.

During the study period, the number of HCQ users with SLE diagnosis was 6,379 in 2004 and increased to 16,578 in 2019 (Fig. 2A and Supplementary Table 4). However, the number of HCQ new users was 1,817 in 2005 and similar or decreased to 1,455 in 2019. The proportion of patients with HCQ among patients with SLE was 63% in 2004 and subsequently increased to 76% in 2019 (Fig. 2B and Supplementary Table 4).

### The trend of daily HCQ dose per ABW

**Figure 3:**
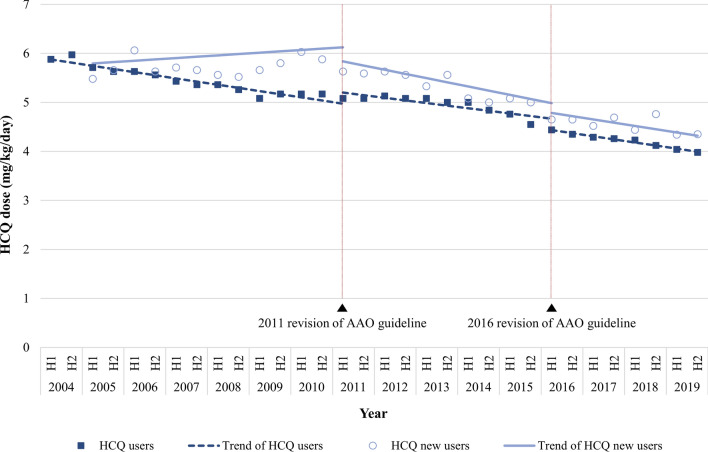
The trend of daily prescription dose per ABW of patients prescribed HCQ for SLE. Dots indicate the median dose per ABW, and the lines show the trend over time for pre- and post-revision of guidelines. ABW, actual body weight; HCQ, hydroxychloroquine; SLE, systemic lupus erythematosus. The unit of analysis is a prescription.

The median daily dose per ABW for patients with HCQ users decreased from 5.88 mg/kg ABW in the first half-year of 2004 to 3.98 mg/kg ABW in the second half-year of 2019 (Fig. 3 and Supplementary Table 5). The trend of daily dose per ABW for HCQ users declined in all periods before and after the 2011 revised guidelines and after the 2016 revised guidelines (Table 2). Median daily dose per ABW by dividing three periods, based on 2011 and 2016 when guidelines were revised, was 5.97 mg (from 2005 to 2010), 5.45 mg (from 2011 to 2015) and 4.59 mg (from 2016 to 2019) for HCQ new users (p-value by Kruskal–Wallis test < 0.0001).

**Table 2:** Interrupted time series analysis parameter results for the daily prescription dose.

	HCQ users			HCQ new users		
	β	95% CI	P-value	β	95% CI	P-value
Intercept (β_0_)	5.94	5.85, 6.03	< 0.0001	5.77	5.54, 6.00	< 0.0001
Baseline slope before 2011 GUIDELINE (β_1_)	− 0.06	− 0.08, − 0.05	< 0.0001	0.03	0.00, 0.06	0.0809
Level change after 2011 GUIDELINE (β_2_)	0.21	0.08, 0.35	0.0039	− 0.18	− 0.50, 0.15	0.2760
Slope change after 2011 GUIDELINE (β_3_)	0.01	− 0.01, 0.03	0.2894	− 0.11	− 0.16, − 0.06	0.0001
Level change after 2016 GUIDELINE (β_4_)	− 0.22	− 0.38, − 0.06	0.0084	− 0.22	− 0.58, 0.15	0.2305
Slope change after 2016 GUIDELINE (β_5_)	− 0.01	− 0.04, 0.02	0.5144	0.02	− 0.05, 0.09	0.5887

The median daily dose per ABW for HCQ new users decreased from 5.45 mg/kg ABW in the first half-year of 2005 to 4.17 mg/kg ABW in the second half-year of 2019 (Fig. 3). Before the 2011 revision of guidelines, the trend of daily dose per ABW for patients as HCQ new users was upward, whereas after the revision, the slope change showed a statistically significant downward trend (− 11% per half-year, 95% CI: − 0.16 ~ − 0.06, p = 0.0001). After the 2016 revision of guidelines, the downward trend continued, and no significant slope change was observed between the 2011–2015 period and after the 2016 period (p-value = 0.5887) (Table 2).

The proportion of patients with HCQ user receiving HCQ doses above 5.0 mg/kg decreased from 66.41% in 2004 to 30.38% in 2019 and decreased from 57.28% in 2005 to 36.70% in 2019 for HCQ new users (Supplementary Table 6).

### The trend of ophthalmologic screening

**Figure 4:**
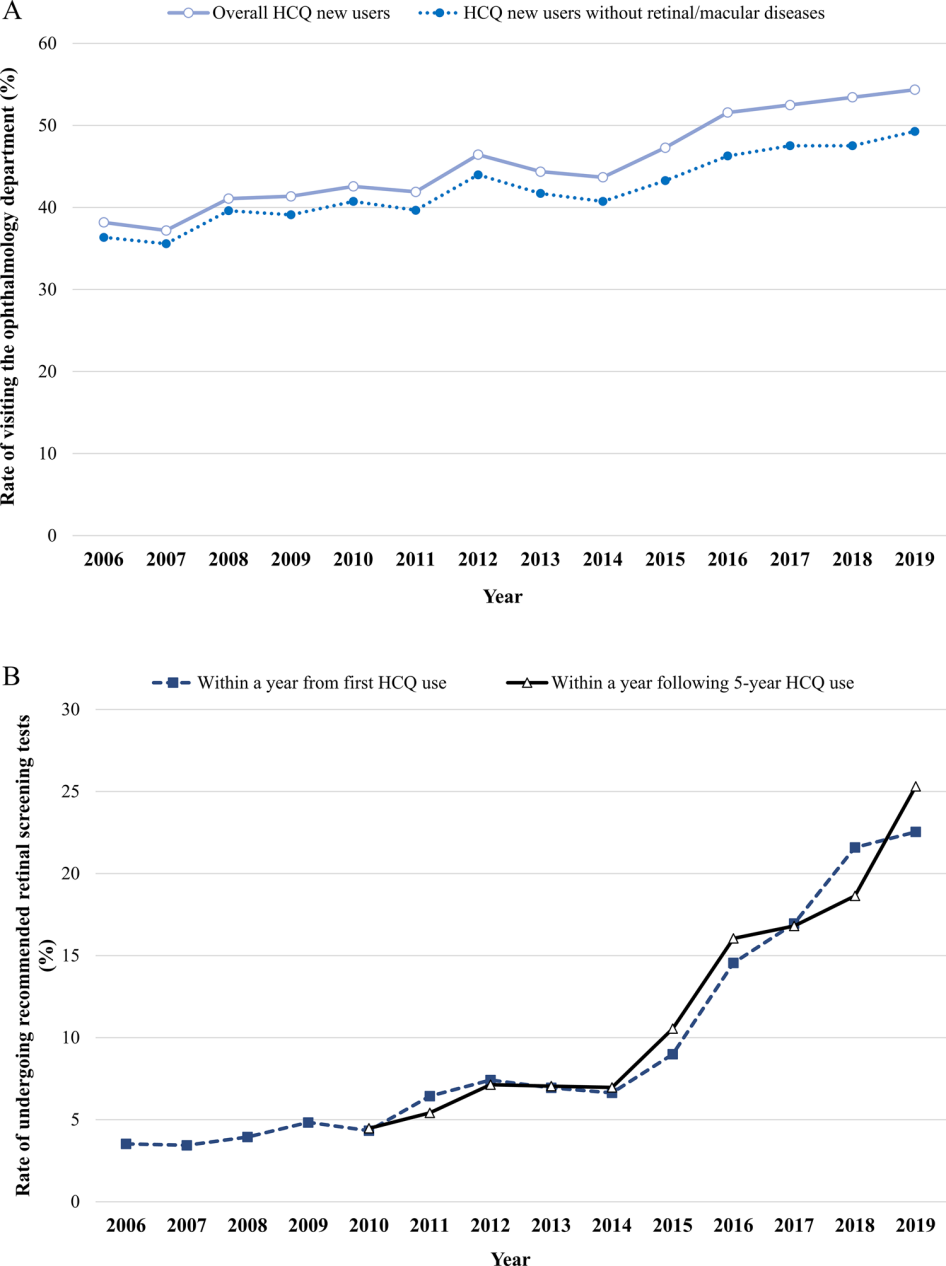
The annual rate of visiting the ophthalmology department (**A**) and undergoing recommended retinal screening tests (**B**) among HCQ new users. ‘Recommended screening tests’ included automated visual fields, SD-OCT, multifocal electroretinogram, and fundus autofluorescence. P-value for trend was < 0.0001.

We determined the changes in the implementation rate of recommended screening tests by year among HCQ new users (Fig. 4). Among overall HCQ new users, the rate of visiting the ophthalmology department was 38.2% in 2006 and increased to 54.4% in 2019. When excluding patients with retinal/macular disease, the rate of patients visiting the ophthalmology department was 36.4% in 2006 and increased to 49.3% in 2019. (Fig. 4A). The rate of undergoing ‘recommended screening tests’ within 1 year from HCQ new use (baseline screening) increased from 3.5% in 2006 to 22.5% in 2019. When we assessed the implementation of recommended screening test during one year following five years of HCQ use, the rate increased from 4.5% in 2010 to 25.3% in 2019 (Fig. 4B).

## Discussion

This study examined the nationwide patterns of HCQ dosing and monitoring of retinal toxicity in patients with SLE following updates to AAO guidelines. The daily dose per ABW showed a continuous declining trend since the revision of guidelines in 2011 and 2016. The implementation rate of retinal screening tests among HCQ new users increased during the study period.

The number of patients with SLE, one of the rare diseases in the South Korean government system, increased from 10,077 in 2004 to 21,786 in 2019 (Supplementary Table 4). A recent study using claims data of NHIS from 2002 to 2015 (washout period was from 2002 to 2004) defined SLE using ICD-10 and ICBP codes^14^. The number of patients with SLE (N = 10,208 in 2005; N = 18,282 in 2015) was comparable with the present study. However, there has been little discussion on the nationwide prevalence of patients using HCQ among SLE patients in South Korea. The present study revealed a high annual proportion, ranging from 63 to 76% from 2004 to 2019. In previous studies, 18.3% of the annual average proportion of patients with SLE were on HCQ in the United States from 2011 to 2015, 47.6% were on HCQ in Germany in 2013, and 69.5% were on HCQ in the UK from 2005 to 2017^15–17^. Given the limited number of studies that have assessed the rate of HCQ utilization in patients with SLE, and the considerable differences among these studies, it would be inappropriate to interpret these results in comparisons with our findings. In the EULAR guideline, HCQ is recommended for all patients with SLE^1^. According to recently published American College of Rheumatology (ACR)- American Academy of Dermatology (AAD)- Rheumatologic Dermatology Society (RDS)- AAO joint statement on HCQ use with respect to retinal toxicity, HCQ should continue until definite diagnostic findings of retinal toxicity^18^. The proportion of HCQ in Korea has increased until recently, and it may be attributable to increased awareness due to the impact of the current guidelines.

According to the drug label, recommended mean daily dose of HCQ ranges from 200-400 mg, whereas the maximum dose should not exceed 6.5 mg/kg/day^19^. In our study, the median daily dose for HCQ users steadily declined from 2004 to 2016. The median daily dose reached below 5 mg/kg in the second half of 2014, which is appropriate for the dose recommended by the 2016 AAO guidelines. A previous study using claim data from the US reported that the HCQ dose per ABW declined from 5.03 mg/kg in 2007 to 4.46 mg/kg HCQ in 2016, and the proportion of dosing above 5.0 mg/kg declined from 49% (2007–2008) to 34% (2015–2016)^9^. Another US study found that the proportions of patients receiving doses above 5.0 mg/kg fell from 38% in 2012 to 30% in 2016^8^. Our results of a declining trend since the revision of the 2011 guidelines are consistent with previous studies. The present study revealed that the proportion of HCQ users receiving doses above 5.0 mg/kg declined from 66.41% in 2004 to 30.38 in 2019 (Supplementary Table 6).

In our analysis, the median daily dose indicating the initial prescriptions among HCQ new users showed a significant declining trend since the revision of the 2011 guidelines. Moreover, the median daily dose was recorded as under 5 mg/kg in 2016, which is appropriate for the 2016 AAO guidelines. After the revision of the guidelines in 2016, the downward trend continued and no significant slope change was observed; however, the daily dose per ABW continued to decrease in both HCQ users and new users.

The present study is meaningful providing real-world patterns of HCQ dosing for over 16 years from 2004 to 2019, including 2011 and 2016 when the AAO guidelines were revised. Namely, our study additionally identified the impact of HCQ dosage management corresponding to the revision of AAO guidelines in 2016 compared to a previous US study covering the period 2007 to 2016^9^. Furthermore, our study reflects the real-world status because we included a total of 22,036 HCQ new users and 28,415 HCQ users with SLE in a nationwide sample.

As excessive daily dose by weight is one of the most critical risk factors for HCQ toxicity, accounting for a patient’s body weight is recommended when prescribing HCQ^7^. In our analysis, we observed the decreasing trend of daily dose per weight, as suggested in the recent guidelines; it can be expected that the risk of HCQ retinal toxicity may have been reduced accordingly. In Korea, five types of HCQ tablets by dose including 100, 150, 200, 300 and 400 mg are available for prescription, while 200 mg HCQ tablets are often prescribed in the USA and Europe^20^. Having multiple types of doses can increase prescription accuracy, which may positively impact the declining trend of daily doses.

Regarding the implementation rate of retinal screening, the present study showed an increasing trend in HCQ new users, recorded at 22.54% in 2019. A recent study in Taiwan revealed an annual screening rate of 1.2% for five years or longer HCQ users and 1.1% for less than five years HCQ users^5^. The Taiwanese study was conducted using the claims database for HCQ users from 1997 to 2007, and defined either SD-OCT, multifocal electroretinograms or automated visual field assessment as screening tests^5^. In the present study, we additionally considered fundus autofluorescence in screening tests, and a one-year assessment period was applied.

The implementation rate of retinal screening increased more than six times since the beginning of the study period, which could have reflected the AAO guidelines in clinical practice. However, the implementation rate was lower than in a European study. In the European Survey for Lupus Patients 2019, 84.3% of patients using HCQ reported they received a baseline examination at the start of HCQ treatment^20^. Although a direct comparison with our study results was not possible because the European Survey did not specify the screening test method, the lower screening rate in the present study suggests the necessity of enhancing awareness among clinicians regarding the importance of retinal screening during HCQ treatment. As such, the following points need consideration while interpreting the difference in screening rate. The lower screening rate in our analysis may be due to our definition of screening rate; whereas the European survey examined ever-implemented retinal screening tests, we assessed the screening rate within one year from the first HCQ prescription and one year after five years of continuous HCQ use. In case of HCQ users, screening rate within one year was 8.99% in 2015. When we assessed during one-year following continuous five years of HCQ use, the screening rate was 10.55% in 2015 (Supplementary Table 7). In addition, the lower screening rate is partly due to lack of coverage by national insurance. In Korea, as fundus autofluorescence and SD-OCT began to be covered by national insurance from 2014 and 2015, respectively, the proportion of screening before 2014 may have been underestimated. As the recommended daily dose based on the AAO guidelines was revised in 2016 to be less than 5 mg/kg, there is a possibility that the screening rate for patients who exceeded the recommended dose temporarily increased.

To the best of our knowledge, this is the first study to examine the trend of daily doses since 2016 and identify the impact of the 2016 AAO guidelines which recommended an HCQ dose of less than 5 mg/kg/day. We could reveal the trend of prescription doses after revising the 2011 and 2016 guidelines, using long-term data of 16 years. Moreover, the daily prescription dose per ABW for each HCQ users and new user was identified using health examination results. To date, no study has suggested the implementation rate of screening tests by year. Also, our study has high generalisability as its strength since it used large-scale population-based data provided by the NHI programme. Furthermore, we used the ICD and the ICBP codes to define SLE. Because SLE is a rare disease, our definition made high validity of the study population.

The present study still has several limitations. First, the patient’s weight information may differ from the actual weight at the prescription time because we used weight information one year before and after the prescription date. Also, patients without weight information were excluded from the analysis. Second, since we could not obtain information on the concurrent drug, the severity of SLE could not be assessed. Third, in terms of the proportion of patients who implemented recommended screening tests, we defined patients as those who implemented at least one of the ‘recommended screening tests’ within a year of the prescription date. The assessment of the screening rate within a year following HCQ use may have contributed the observation of the lower rate. However, there is also a possibility of overestimation due to the limitation of accuracy when applying clinical retinal examination techniques suggested by the AAO guidelines to insurance claims codes. Fourth, due to the nature of the claims database, it was difficult to examine if patients have retinal dystrophy or significant degeneration, which were retinal/macular risk factors mentioned in the 2016 AAO guidelines. We defined comorbid retinal/macular disease as at least one diagnosis of ‘disorders of choroid and retina’ (ICD-10, H3) within a year from the first prescription date. Finally, in our database, we could not identify whether dose reduction was due to the incidence of retinopathy/ HCQ toxicity or changes in SLE disease activity. Furthermore, a recent study showed reduction of HCQ following AAO guidelines or low disease activity is associated with SLE flares^21^. Thus, it is important to determine dose reduction considering the risk of flares and to carefully monitor the patient’s disease activity. Further study assessing dose reduction, SLE flares, and incidence of retinal toxicity are required.

## Conclusion

We found that HCQ dosing management was adequate based on the revised AAO guidelines for HCQ in patients with SLE in Korea. However, despite a recent increase, the rate of implemented retinal screening tests remains low considering recommendations in the guidelines. It suggests the need to enhance awareness of retinal screening in clinical settings. Further studies are needed to determine whether a decrease in the daily prescription dose of HCQ and an increase in retinal screening reduce incidences of HCQ retinopathy, while considering the impact of HCQ reduction on retinal toxicity and risk of SLE flares.

## Supplementary Information

Supplementary Information.

## Data Availability

The datasets generated and/or analysed during the current study are not publicly available because all data have been deposited in the National Health Insurance Service (NHIS). Data are, however, available from S.Y.J upon reasonable request and with permission of the NHIS. To protect the privacy and public interests, de-identified raw data is provided by the NHIS, and access to the data is available only in designated research centres in the Republic of Korea.
