# Supplementary material for: Nationwide patterns of hydroxychloroquine dosing and monitoring of retinal toxicity in patients with systemic lupus erythematosus
Source: Sci Rep. 2023 May 4;13:7270. doi: 10.1038/s41598-023-34022-0 (PMC10160043; doi:10.1038/s41598-023-34022-0)
Supplement: Supplementary file 1 — Supplementary Information. [file 41598_2023_34022_MOESM1_ESM.pdf]

Supplementary Tables Legends

**Supplementary Table 1**	Definition of retinal/macular disease by ICD-10 code
**Supplementary Table 2**	Procedure codes of retinal screening tests from the Health Insurance Review and Assessment Service (HIRA)
**Supplementary Table 3**	Characteristics of patients prescribed HCQ for SLE by year
**Supplementary Table 4**	The number of patients with SLE and HCQ users (%) by year
**Supplementary Table 5**	The median daily dose per ABW for patients prescribed HCQ for SLE by year
**Supplementary Table 6**	The proportion of patients receiving HCQ doses above 5.0 mg/kg
**Supplementary Table 7**	The number of HCQ new users and patients who implemented recommended screening tests among HCQ new users by year

Supplementary Table 1. Definition of retinal/macular disease by ICD-10 code

Code range	Section description
H3	Disorders of choroid and retina
H30	Chorioretinal inflammation
H30.0	Focal chorioretinal inflammation
H30.1	Disseminated chorioretinal inflammation
H30.2	Posterior cyclitis
H30.8	Other chorioretinal inflammation
H30.9	Unspecified chorioretinal inflammation
H31	Other disorders of choroid
H31.0	Chorioretinal scar
H31.1	Choroidal degeneration
H31.2	Hereditary choroidal dystrophy
H31.3	Choroidal haemorrhage and rupture
H31.4	Choroidal detachment
H31.8	Other specified disorders of choroid
H31.9	Unspecified disorder of choroid
H32	Chorioretinal disorders in diseases classified elsewhere
H33	Retinal detachments and breaks
H33.0	Retinal detachment with retinal break
H33.1	Retinoschisis and retinal cysts
H31.2	Serious retinal detachment
H33.3	Retinal breaks without detachment
H33.4	Traction detachment of retina
H33.8	Other retinal detachments
H34	Retinal vascular occlusions
H34.0	Transient retinal artery occlusion
H34.1	Central retinal artery occlusion

H34.2	Other retinal artery occlusions
H34.8	Other retinal vascular occlusions
H34.9	Unspecified retinal vascular occlusion
H35	Other retinal disorders
H35.0	Background retinopathy and retinal vascular changes
H35.1	Retinopathy of prematurity
H35.2	Other non-diabetic proliferative retinopathy
H35.3	Degeneration of macula and posterior pole
H35.4	Peripheral retinal degeneration
H35.5	Hereditary retinal dystrophy
H35.6	Retinal haemorrhage
H35.7	Separation of retinal layers
H35.8	Other specified retinal disorders
H35.9	Unspecified retinal disorder
H36	Retinal disorders in diseases classified elsewhere

ICD, International Classification of Diseases.

Supplementary Table 2. Procedure codes of retinal screening tests from the Health Insurance Review and Assessment Service (HIRA)

Retinal screening tests from guideline		Procedure code descriptions from HIRA	Procedure codes from HIRA
Primary screening tests	Automated visual field assessment	Automated visual field assessment	E6691 (2004~2019)
	Spectral-domain optical coherence tomography	Optical coherence tomography	EZ796 (2015~2019)
Other recommended tests	Multifocal electroretinography	Electroretinography,<br>Standard electroretinography	E6684 (2004~2016),<br>E6685 (2017~2019)
	Fundus autofluorescence	Fundus autofluorescence	E6675 (2014~2019)

Supplementary Table 3. Characteristics of patients prescribed HCQ for SLE by year

3-A. Characteristics of HCQ users from 2004 to 2019

HCQ users, Year (N)	2004 (6,379)	2005 (7,140)	2006 (7,878)	2007 (8,388)	2008 (9,020)	2009 (9,807)	2010 (9,647)	2011 (10,383)	2012 (11,227)	2013 (12,165)	2014 (13,074)	2015 (13,683)	2016 (14,562)	2017 (15,287)	2018 (16,058)	2019 (16,578)
Age																
Median (min, max)	37 (5, 84)	37 (8, 85)	38 (4, 85)	39 (5, 94)	40 (6, 90)	40 (7, 90)	40 (1, 91)	41 (8, 90)	42 (5, 91)	43 (6, 92)	43 (7, 92)	44 (1, 93)	44 (8, 94)	45 (9, 95)	46 (1, 96)	46 (6, 104)
Mean (SD)	38.3 (12.31)	38.83 (12.46)	39.3 (12.46)	40.08 (12.55)	40.66 (12.71)	41.25 (12.87)	41.45 (12.98)	42.09 (13.09)	42.58 (13.34)	43.13 (13.52)	43.71 (13.66)	44.21 (13.83)	44.87 (14.06)	45.26 (14.22)	45.8 (14.36)	46.18 (14.46)
<40, n (%)	3640 (57.06)	4003 (56.06)	4268 (54.18)	4346 (51.81)	4501 (49.9)	4689 (47.81)	4493 (46.57)	4601 (44.31)	4755 (42.35)	4981 (40.95)	5157 (39.44)	5223 (38.17)	5318 (36.52)	5507 (36.02)	5566 (34.66)	5558 (33.53)
≥40, n (%)	2739 (42.94)	3137 (43.94)	3610 (45.82)	4042 (48.19)	4519 (50.1)	5118 (52.19)	5154 (53.43)	5782 (55.69)	6472 (57.65)	7184 (59.05)	7917 (60.56)	8460 (61.83)	9244 (63.48)	9780 (63.98)	10492 (65.34)	11020 (66.47)
Sex																
Men, n (%)	497 (7.79)	567 (7.94)	655 (8.31)	695 (8.29)	741 (8.22)	790 (8.06)	759 (7.87)	846 (8.15)	927 (8.26)	1061 (8.72)	1149 (8.79)	1236 (9.03)	1315 (9.03)	1411 (9.23)	1492 (9.29)	1568 (9.46)
Women, n (%)	5882 (92.21)	6573 (92.06)	7223 (91.69)	7693 (91.71)	8279 (91.78)	9017 (91.94)	8888 (92.13)	9537 (91.85)	10300 (91.74)	11104 (91.28)	11925 (91.21)	12447 (90.97)	13247 (90.97)	13876 (90.77)	14566 (90.71)	15010 (90.54)
Risk factor																
CKD (≥stage 3), n (%)	7 (0.11)	18 (0.25)	30 (0.38)	29 (0.35)	49 (0.54)	52 (0.53)	57 (0.59)	91 (0.88)	104 (0.93)	154 (1.27)	175 (1.34)	184 (1.34)	212 (1.46)	232 (1.52)	278 (1.73)	328 (1.98)
Retinal/macular disease, n (%)	329 (5.16)	509 (7.13)	615 (7.81)	608 (7.25)	628 (6.96)	772 (7.87)	774 (8.02)	993 (9.56)	1231 (10.96)	1447 (11.89)	1761 (13.47)	2314 (16.91)	3013 (20.69)	3791 (24.8)	4315 (26.87)	5115 (30.85)

3-B. Characteristics of HCQ users with ABW information from 2004 to 2019

HCQ users with ABW information, Year (N)	2004 (1,182)	2005 (1,878)	2006 (2,215)	2007 (2,690)	2008 (3,254)	2009 (3,955)	2010 (4,136)	2011 (4,692)	2012 (5,207)	2013 (5,811)	2014 (6,469)	2015 (6,986)	2016 (7,689)	2017 (8,298)	2018 (7,969)	2019 (4,651)
Age																
Median (min, max)	42.5 (19, 80)	44 (18, 81)	45 (19, 82)	44 (18, 94)	45 (19, 90)	45 (19, 85)	46 (19, 86)	46 (19, 87)	46 (19, 88)	47 (18, 89)	47 (19, 87)	47 (19, 88)	48 (18, 86)	48 (19, 95)	49 (20, 96)	49 (21, 88)
Mean (SD)	41.87 (11.42)	43.18 (11.76)	43.9 (11.73)	44.23 (11.99)	44.84 (11.89)	45.6 (11.79)	45.82 (11.65)	46.32 (11.65)	46.84 (11.66)	47.29 (11.74)	47.62 (11.86)	48.02 (11.98)	48.6 (12.05)	48.83 (12.13)	49.41 (12.12)	49.26 (12.05)
<40, n (%)	475 (40.19)	688 (36.63)	769 (34.72)	925 (34.39)	1047 (32.18)	1171 (29.61)	1148 (27.76)	1237 (26.36)	1283 (24.64)	1373 (23.63)	1518 (23.47)	1596 (22.85)	1633 (21.24)	1756 (21.16)	1624 (20.38)	979 (21.05)
≥40, n (%)	707 (59.81)	1190 (63.37)	1446 (65.28)	1765 (65.61)	2207 (67.82)	2784 (70.39)	2988 (72.24)	3455 (73.64)	3924 (75.36)	4438 (76.37)	4951 (76.53)	5390 (77.15)	6056 (78.76)	6542 (78.84)	6345 (79.62)	3672 (78.95)
Sex																
Men, n (%)	117 (9.9)	162 (8.63)	215 (9.71)	246 (9.14)	299 (9.19)	341 (8.62)	332 (8.03)	381 (8.12)	417 (8.01)	496 (8.54)	564 (8.72)	611 (8.75)	668 (8.69)	719 (8.66)	706 (8.86)	468 (10.06)
Women, n (%)	1065 (90.1)	1716 (91.37)	2000 (90.29)	2444 (90.86)	2955 (90.81)	3614 (91.38)	3804 (91.97)	4311 (91.88)	4790 (91.99)	5315 (91.46)	5905 (91.28)	6375 (91.25)	7021 (91.31)	7579 (91.34)	7263 (91.14)	4183 (89.94)
Risk factor																
CKD (≥stage 3), n (%)	1 (0.08)	2 (0.11)	5 (0.23)	3 (0.11)	6 (0.18)	10 (0.25)	11 (0.27)	26 (0.55)	22 (0.42)	45 (0.77)	60 (0.93)	62 (0.89)	74 (0.96)	95 (1.14)	115 (1.44)	78 (1.68)
Retinal/macular disease, n (%)	59 (4.99)	129 (6.87)	177 (7.99)	204 (7.58)	222 (6.82)	324 (8.19)	336 (8.12)	482 (10.27)	631 (12.12)	744 (12.8)	923 (14.27)	1262 (18.06)	1682 (21.88)	2164 (26.08)	2256 (28.31)	1498 (32.21)
ABW (kg)*																
Median (min, max)	54 (36, 103)	54 (36, 103)	55 (35, 102)	55 (32, 104)	55 (32, 114)	55 (28, 114)	55 (28, 114)	55 (33, 111)	55 (31, 110)	55 (31, 111)	55 (28, 121)	55 (28, 121)	55 (30, 126)	55.9 (28, 126)	56 (28, 131)	56.2 (28, 131)

Mean (SD)	55.73 (8.89)	55.89 (8.81)	56.17 (8.86)	56.2 (9.02)	56.26 (9.2)	56.14 (9.02)	56.1 (8.95)	56.47 (9.28)	56.45 (9.46)	56.53 (9.46)	56.6 (9.65)	56.8 (9.82)	57.01 (9.95)	57.46 (10.29)	57.73 (10.42)	58.13 (10.73)
0< ≤45, n (%)	98 (8.29)	152 (8.09)	170 (7.67)	222 (8.25)	262 (8.05)	325 (8.22)	327 (7.91)	367 (7.82)	439 (8.43)	483 (8.31)	540 (8.35)	589 (8.43)	614 (7.99)	607 (7.32)	548 (6.88)	323 (6.94)
45< ≤55, n (%)	568 (48.05)	883 (47.02)	1,029 (46.46)	1,204 (44.76)	1,462 (44.93)	1,758 (44.45)	1,865 (45.09)	2,075 (44.22)	2,259 (43.38)	2,515 (43.28)	2,825 (43.67)	3,021 (43.24)	3,258 (42.37)	3,373 (40.65)	3,144 (39.45)	1,760 (37.84)
55< ≤65, n (%)	365 (30.88)	593 (31.58)	698 (31.51)	879 (32.68)	1,079 (33.16)	1,341 (33.91)	1,395 (33.73)	1,563 (33.31)	1,741 (33.44)	1,965 (33.82)	2,125 (32.85)	2,230 (31.92)	2,513 (32.68)	2,780 (33.5)	2,698 (33.86)	1,597 (34.34)
>65, n (%)	151 (12.77)	250 (13.31)	318 (14.36)	385 (14.31)	451 (13.86)	531 (13.43)	549 (13.27)	687 (14.64)	768 (14.75)	848 (14.59)	979 (15.13)	1,146 (16.4)	1,304 (16.96)	1,538 (18.53)	1,579 (19.81)	971 (20.88)

3-C. Characteristics of HCQ new users from 2005 to 2019

HCQ new users, Year (N)	2005 (1,817)	2006 (1,629)	2007 (1,368)	2008 (1,407)	2009 (1,548)	2010 (1,181)	2011 (1,294)	2012 (1,325)	2013 (1,387)	2014 (1,480)	2015 (1,388)	2016 (1,581)	2017 (1,570)	2018 (1,606)	2019 (1,455)
Age															
Median (min, max)	37 (9, 84)	37 (4, 84)	38 (9, 94)	38 (9, 86)	39 (10, 90)	38 (1, 86)	39 (8, 86)	39 (5, 84)	40 (7, 90)	40 (7, 86)	41 (1, 89)	43 (8, 91)	42 (10, 95)	42 (1, 92)	41 (6, 104)
Mean (SD)	38.93 (13.57)	38.45 (13.33)	39.46 (14.1)	39.41 (14.02)	39.97 (14.29)	38.86 (14.78)	39.65 (14.82)	39.43 (15.42)	40.54 (15.59)	41.34 (15.97)	41.63 (16.09)	43.24 (17.09)	43.35 (17.11)	43.78 (17.06)	43.25 (17.47)
<40, n (%)	1026 (56.47)	898 (55.13)	737 (53.87)	755 (53.66)	807 (52.13)	653 (55.29)	672 (51.93)	680 (51.32)	680 (49.03)	704 (47.57)	651 (46.9)	683 (43.2)	714 (45.48)	712 (44.33)	661 (45.43)
≥40, n (%)	791 (43.53)	731 (44.87)	631 (46.13)	652 (46.34)	741 (47.87)	528 (44.71)	622 (48.07)	645 (48.68)	707 (50.97)	776 (52.43)	737 (53.1)	898 (56.8)	856 (54.52)	894 (55.67)	794 (54.57)
Sex															
Men, n (%)	174 (9.58)	156 (9.58)	136 (9.94)	137 (9.74)	137 (8.85)	83 (7.03)	133 (10.28)	142 (10.72)	175 (12.62)	167 (11.28)	177 (12.75)	178 (11.26)	198 (12.61)	196 (12.2)	188 (12.92)
Women, n (%)	1643 (90.42)	1473 (90.42)	1232 (90.06)	1270 (90.26)	1411 (91.15)	1098 (92.97)	1161 (89.72)	1183 (89.28)	1212 (87.38)	1313 (88.72)	1211 (87.25)	1403 (88.74)	1372 (87.39)	1410 (87.8)	1267 (87.08)
Risk factor															
CKD (≥stage 3), n (%)	10 (0.55)	8 (0.49)	11 (0.8)	18 (1.28)	10 (0.65)	8 (0.68)	15 (1.16)	16 (1.21)	31 (2.24)	28 (1.89)	21 (1.51)	40 (2.53)	37 (2.36)	33 (2.05)	41 (2.82)
Retinal/macular disease, n (%)	115 (6.33)	123 (7.55)	116 (8.48)	116 (8.24)	117 (7.56)	87 (7.37)	130 (10.05)	148 (11.17)	187 (13.48)	209 (14.12)	258 (18.59)	304 (19.23)	398 (25.35)	423 (26.34)	422 (29)

3-D. Characteristics of HCQ new users with ABW information from 2005 to 2019

HCQ new users with ABW information, Year (N)	2005 (1,043)	2006 (972)	2007 (829)	2008 (873)	2009 (958)	2010 (727)	2011 (787)	2012 (765)	2013 (806)	2014 (829)	2015 (760)	2016 (807)	2017 (729)	2018 (643)	2019 (246)
Age															
Median (min, max)	38 (14, 76)	39 (10, 78)	40 (10, 94)	40 (12, 80)	42 (14, 80)	41 (16, 79)	42 (16, 82)	43 (16, 80)	44 (16, 83)	44 (18, 86)	46 (17, 83)	48 (18, 85)	49 (20, 95)	48 (21, 85)	48 (23, 88)
Mean (SD)	39.78 (12.35)	39.98 (12.36)	40.42 (13.22)	40.83 (12.64)	42.44 (13.09)	42.56 (12.79)	42.41 (12.92)	43.32 (13.34)	44.54 (13.34)	45.32 (13.79)	46.52 (12.96)	48.55 (13.51)	48.67 (14)	48.99 (13.56)	48.64 (14.49)
<40, n (%)	553 (53.02)	489 (50.31)	414 (49.94)	418 (47.88)	417 (43.53)	322 (44.29)	340 (43.2)	296 (38.69)	277 (34.37)	276 (33.29)	227 (29.87)	201 (24.91)	196 (26.89)	167 (25.97)	69 (28.05)
≥40, n (%)	490 (46.98)	483 (49.69)	415 (50.06)	455 (52.12)	541 (56.47)	405 (55.71)	447 (56.8)	469 (61.31)	529 (65.63)	553 (66.71)	533 (70.13)	606 (75.09)	533 (73.11)	476 (74.03)	177 (71.95)
Sex															
Men, n (%)	88 (8.44)	84 (8.64)	70 (8.44)	79 (9.05)	80 (8.35)	38 (5.23)	81 (10.29)	77 (10.07)	102 (12.66)	97 (11.7)	93 (12.24)	89 (11.03)	84 (11.52)	83 (12.91)	29 (11.79)
Women, n (%)	955 (91.56)	888 (91.36)	759 (91.56)	794 (90.95)	878 (91.65)	689 (94.77)	706 (89.71)	688 (89.93)	704 (87.34)	732 (88.3)	667 (87.76)	718 (88.97)	645 (88.48)	560 (87.09)	217 (88.21)
Risk factor															
CKD (≥stage 3), n	1 (0.1)	0 (0)	3 (0.36)	6 (0.69)	5 (0.52)	2 (0.28)	4 (0.51)	3 (0.39)	10 (1.24)	12 (1.45)	8 (1.05)	18 (2.23)	14 (1.92)	10 (1.56)	9 (3.66)

(%)															
Retinal/macular disease, n (%)	70 (6.71)	74 (7.61)	70 (8.44)	75 (8.59)	67 (6.99)	66 (9.08)	81 (10.29)	82 (10.72)	108 (13.4)	131 (15.8)	151 (19.87)	164 (20.32)	199 (27.3)	184 (28.62)	81 (32.93)
ABW (kg)*															
Median (min, max)	55 (34, 106)	55 (32, 102)	55 (28, 126)	55 (33, 104.7)	55 (33, 97)	55 (36, 109)	56 (38, 110)	55.9 (35, 113)	56 (37, 103)	56 (32, 119)	56 (37, 104)	56 (35, 112.2)	56.5 (33.6, 122)	56.5 (37.8, 101.4)	55.4 (35.9, 124.7)
Mean (SD)	56.31 (9.25)	56.17 (8.94)	56.5 (9.64)	56.62 (9.83)	56.27 (8.88)	56.13 (9.19)	57.89 (10.52)	57.44 (10.85)	57.64 (10.1)	57.72 (11.15)	58.4 (10.52)	57.45 (10.17)	58.21 (10.52)	58.27 (9.96)	57.84 (10.04)
0< ≤45, n (%)	92 (8.82)	78 (8.02)	68 (8.2)	72 (8.25)	71 (7.41)	64 (8.8)	47 (5.97)	67 (8.76)	63 (7.82)	68 (8.2)	46 (6.05)	60 (7.43)	39 (5.35)	33 (5.13)	17 (6.91)
45< ≤55, n (%)	452 (43.34)	418 (43)	359 (43.31)	388 (44.44)	442 (46.14)	325 (44.7)	333 (42.31)	314 (41.05)	319 (39.58)	344 (41.5)	311 (40.92)	326 (40.4)	293 (40.19)	247 (38.41)	98 (39.84)
55< ≤65, n (%)	350 (33.56)	337 (34.67)	279 (33.66)	273 (31.27)	307 (32.05)	248 (34.11)	262 (33.29)	236 (30.85)	278 (34.49)	255 (30.76)	236 (31.05)	267 (33.09)	259 (35.53)	227 (35.3)	84 (34.15)
>65, n (%)	149 (14.29)	139 (14.3)	123 (14.84)	140 (16.04)	138 (14.41)	90 (12.38)	145 (18.42)	148 (19.35)	146 (18.11)	162 (19.54)	167 (21.97)	154 (19.08)	138 (18.93)	136 (21.15)	47 (19.11)

CKD, chronic kidney disease; ABW, actual body weight.

*ABW information was obtained based on the first prescription date for each year of patients with ABW information.

Supplementary Table 4. The number of patients with SLE and HCQ users (%) by year

Year	Prevalent SLE			Incident SLE		
	Population	HCQ users		Population	HCQ users	
	N	n	(%)	N	n	(%)
2004	10,077	6,379	(63.3)	-	-	-
2005	10,551	7,140	(67.7)	2,793	1,817	(65.1)
2006	11,500	7,878	(68.5)	2,440	1,629	(66.8)
2007	12,322	8,388	(68.1)	2,166	1,368	(63.2)
2008	13,103	9,020	(68.8)	1,990	1,407	(70.7)
2009	14,047	9,807	(69.8)	2,071	1,548	(74.8)
2010	13,422	9,647	(71.9)	1,391	1,181	(84.9)
2011	14,372	10,383	(72.2)	1,497	1,294	(86.4)
2012	15,308	11,227	(73.3)	1,479	1,325	(89.6)
2013	16,331	12,165	(74.5)	1,584	1,387	(87.6)
2014	17,510	13,074	(74.7)	1,720	1,480	(86.1)
2015	18,384	13,683	(74.4)	1,929	1,388	(72.0)
2016	19,578	14,562	(74.4)	2,165	1,581	(73.0)
2017	20,533	15,287	(74.5)	2,127	1,570	(73.8)
2018	21,150	16,058	(75.9)	1,875	1,606	(85.7)
2019	21,786	16,578	(76.1)	1,669	1,455	(87.2)

Supplementary Table 5. The median daily dose per ABW for patients prescribed HCQ for SLE by year

Year		HCQ dose (mg/kg/day)	
		HCQ users	HCQ new users
2004	H1	5.88 (4-7.27)	-
	H2	5.97 (4.08-7.41)	-
2005	H1	5.71 (4-7.14)	5.48 (4-6.78)
	H2	5.63 (3.92-7.14)	5.66 (3.92-7.14)
2006	H1	5.63 (3.85-7.14)	6.06 (4.08-7.27)
	H2	5.56 (3.77-7.14)	5.63 (3.92-7.02)
2007	H1	5.43 (3.77-7.02)	5.71 (4-7.14)
	H2	5.36 (3.7-7.02)	5.66 (3.7-7.27)
2008	H1	5.36 (3.7-7.02)	5.56 (3.77-7.14)
	H2	5.26 (3.7-7.02)	5.52 (3.92-7.02)
2009	H1	5.08 (3.7-7.02)	5.66 (3.7-7.41)
	H2	5.17 (3.7-6.9)	5.8 (4-7.14)
2010	H1	5.17 (3.7-7.02)	6.03 (3.92-7.27)
	H2	5.17 (3.7-7.02)	5.88 (4-7.27)
2011	H1	5.08 (3.7-6.98)	5.63 (3.77-7.27)
	H2	5.08 (3.7-6.9)	5.59 (3.85-7.14)
2012	H1	5.13 (3.7-6.9)	5.63 (3.85-7.14)
	H2	5.08 (3.64-6.9)	5.56 (4-7.14)
2013	H1	5.08 (3.64-6.9)	5.33 (3.77-6.9)
	H2	5 (3.64-6.9)	5.56 (3.85-7.08)
2014	H1	5 (3.64-6.9)	5.08 (3.7-6.78)
	H2	4.84 (3.64-6.67)	5 (3.85-6.78)
2015	H1	4.76 (3.64-6.45)	5.08 (3.7-6.78)
	H2	4.55 (3.57-6.35)	5 (3.64-6.67)
2016	H1	4.44 (3.57-6.15)	4.65 (3.57-6.52)

	H2	4.35 (3.53-6.12)	4.65 (3.57-6.29)
2017	H1	4.29 (3.51-5.97)	4.52 (3.54-6.08)
	H2	4.26 (3.5-5.81)	4.69 (3.64-6.02)
2018	H1	4.23 (3.49-5.74)	4.44 (3.59-6.02)
	H2	4.12 (3.44-5.56)	4.76 (3.57-6.03)
2019	H1	4.04 (3.39-5.39)	4.34 (3.58-5.66)
	H2	3.98 (3.32-5.15)	4.35 (3.63-5.56)

Values are shown as median (IQR).

The unit of analysis is a prescription.

Supplementary Table 6. The proportion of patients receiving HCQ doses above 5.0 mg/kg

Year	HCQ users (%)	HCQ new users (%)
2004	66.41	-
2005	62.83	57.28
2006	61.9	66.38
2007	57.51	63.04
2008	55.81	63.60
2009	53.35	61.16
2010	52.68	64.46
2011	52.26	60.10
2012	51.62	59.98
2013	51.51	57.87
2014	49.39	51.80
2015	46.42	51.63
2016	42.89	45.70
2017	39.13	43.15
2018	35.58	42.77
2019	30.38	36.70

Supplementary Table 7. The number of HCQ new users and patients who underwent recommended screening tests among HCQ new users by year

Year	HCQ new users	Recommended screening tests	Primary screening tests		Other recommended tests		HCQ 5-year users	Recommended screening tests	Primary screening tests		Other recommended tests	
			Automated visual field assessment	SD-OCT	Multifocal electro-retinography	Fundus autofluorescence			Automated visual field assessment	SD-OCT	Multifocal electro-retinography	Fundus autofluorescence
2006	1,817	64 (3.52)	64 (3.52)									
2007	1,629	56 (3.44)	53 (3.25)		3 (0.18)							
2008	1,368	54 (3.95)	53 (3.87)		2 (0.15)							
2009	1407	68 (4.84)	66 (4.69)		4 (0.28)							
2010	1,548	67 (4.33)	67 (4.33)		1 (0.06)		872	39 (4.47)	39 (4.47)		2 (0.23)	
2011	1,181	76 (6.44)	74 (6.27)		4 (0.34)		794	43 (5.42)	43 (5.42)		1 (0.13)	
2012	1,294	96 (7.42)	95 (7.34)		4 (0.31)		645	46 (7.13)	45 (6.98)		2 (0.31)	
2013	1,325	92 (6.94)	92 (6.94)		3 (0.23)		710	50 (7.04)	50 (7.04)		1 (0.14)	
2014	1,387	92 (6.63)	89 (6.42)		3 (0.22)	5 (0.36)	804	56 (6.97)	54 (6.72)			7 (0.87)
2015	1,480	133 (8.99)	109 (7.36)	51 (3.45)	1 (0.07)	34 (2.3)	711	75 (10.55)	63 (8.86)	41 (5.77)	1 (0.14)	33 (4.64)
2016	1,388	202 (14.55)	131 (9.44)	122 (8.79)	1 (0.07)	52 (3.75)	785	126 (16.05)	97 (12.36)	89 (11.34)	3 (0.38)	47 (5.99)
2017	1,581	268 (16.95)	195 (12.33)	175 (11.07)	2 (0.13)	89 (5.63)	821	138 (16.81)	92 (11.21)	104 (12.67)		51 (6.21)
2018	1,570	339 (21.59)	215 (13.69)	274 (17.45)	8 (0.51)	139 (8.85)	869	162 (18.64)	123 (14.15)	122 (14.04)	4 (0.46)	68 (7.83)
2019	1,606	362 (22.54)	219 (13.64)	308 (19.18)	5 (0.31)	131 (8.16)	893	226 (25.31)	154 (17.25)	192 (21.5)	1 (0.11)	108 (12.09)

'Recommended screening tests' include automated visual fields, SD-OCT, multifocal electroretinogram and fundus autofluorescence.

Years in this table are based on when screening rates were evaluated.
